# Supplementary material for: Gene signature driving invasive mucinous adenocarcinoma of the lung
Source: EMBO Mol Med. 2017 Mar 2;9(4):462–81. doi: 10.15252/emmm.201606711 (PMC5376761; doi:10.15252/emmm.201606711)
Supplement: Supplementary file 1 — Appendix [file EMMM-9-462-s001.pdf]

Gene signature driving invasive mucinous adenocarcinoma of the lung

Minzhe Guo, Koichi Tomoshige, Michael Meister, Thomas Muley, Takuya Fukazawa, Tomoshi Tsuchiya, Rebekah Karns, Arne Warth, Iris M. Fink-Baldauf, Takeshi Nagayasu, Yoshio Naomoto, Yan Xu, Marcus A. Mall and Yutaka Maeda

Appendix

Figures S1 – S3

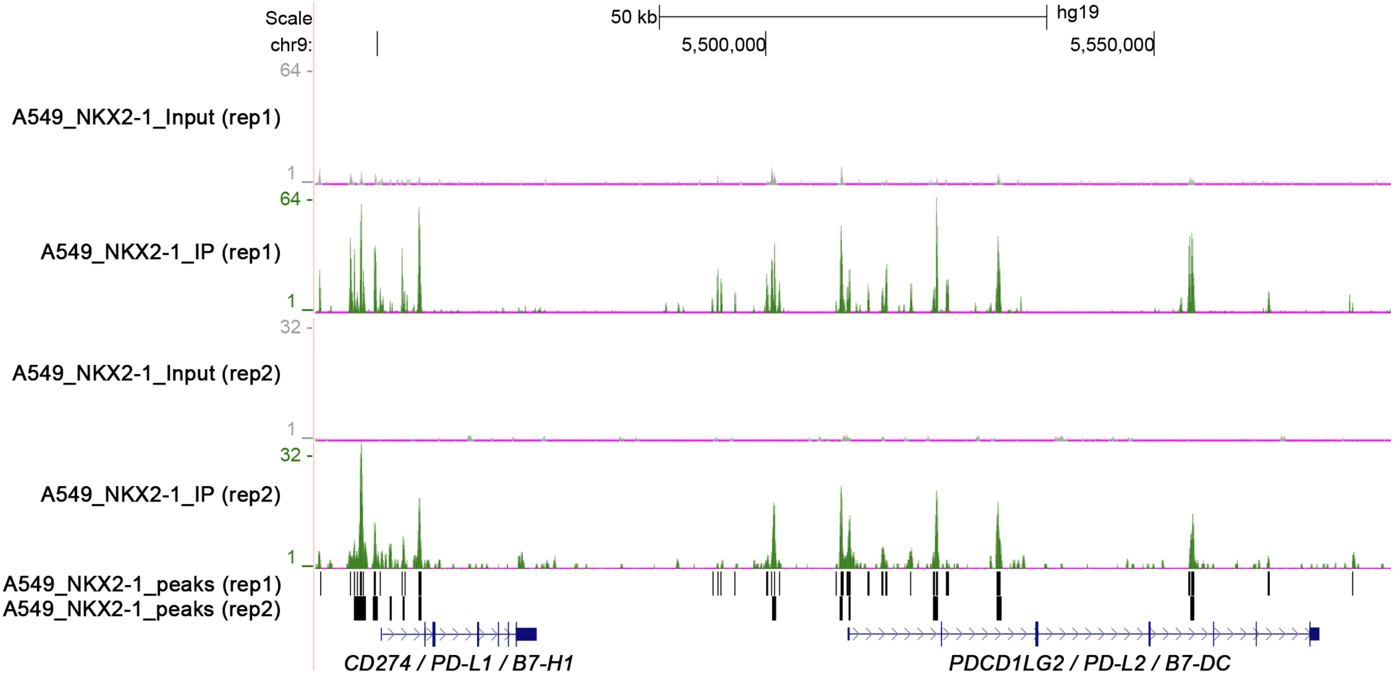

Appendix Figure S1. NKX2-1 (also known as TTF-1) bound to multiple sites in the locus of *PD-L1* (also known as *CD274* or *B7-H1*) and *PD-L2* (*PDCD1LG2*).

Shown is a UCSC genome browser view of ChIP-seq indicating NKX2-1 binding sites (A549-NKX2-1_IP) along with Input (A549_NKX2-1_Input). Two biological replicates data are shown. ChIP-seq was performed using NKX2-1 (TTF-1) antibody and chromatin from A549 cells infected with *Nkx2-1*-expressing lentivirus as previously reported (Maeda et al, 2012). Chr, chromosome.

A

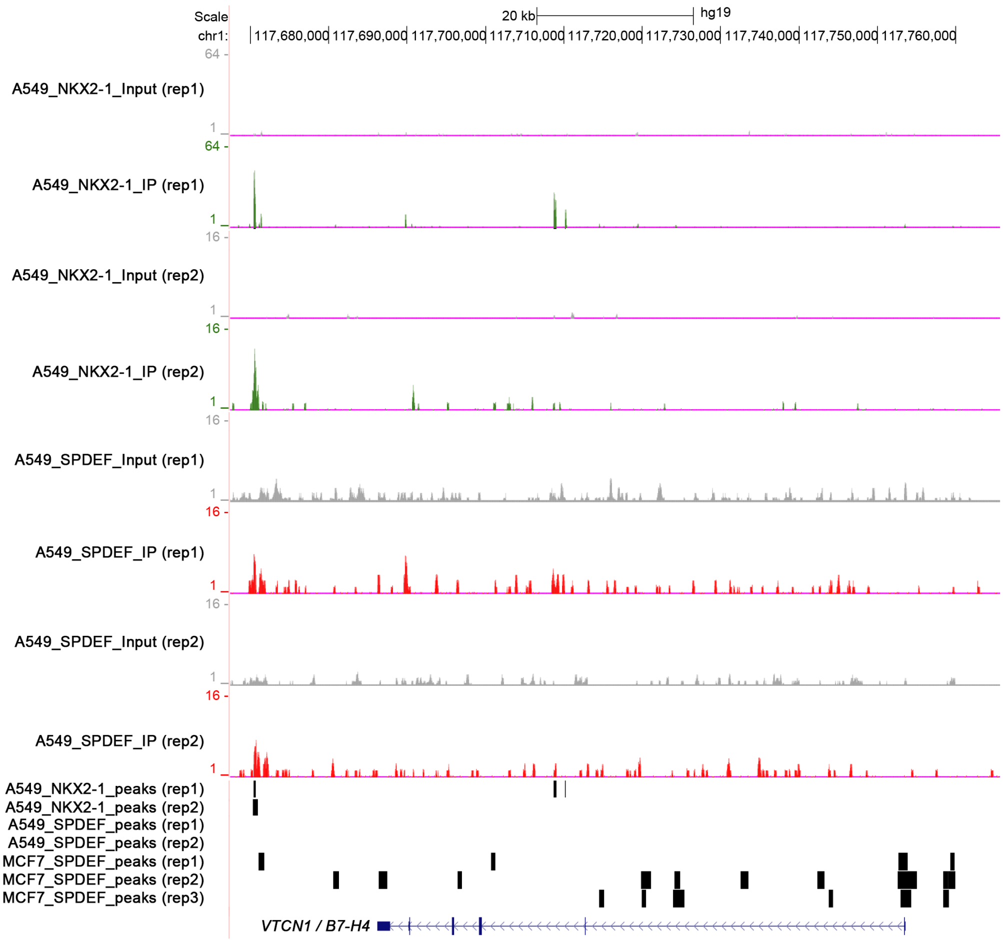

B

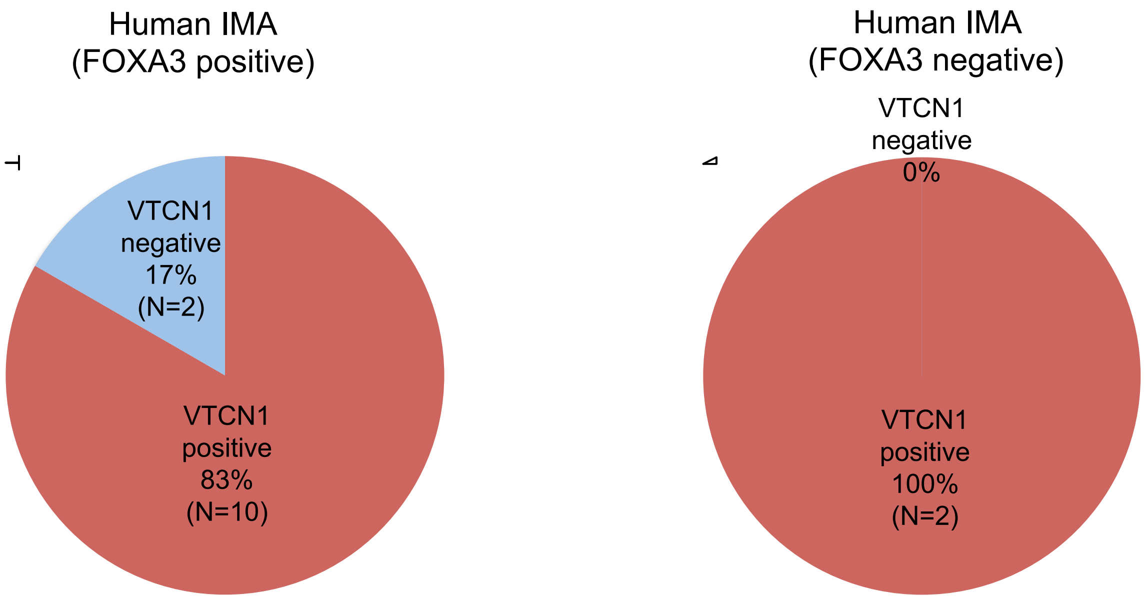

Appendix Figure S2. VTCN1 is expressed in human IMA independently of SPDEF or FOXA3.

A Shown is a UCSC genome browser view of ChIP-seq indicating NKX2-1 (A549_NKX2-1_IP) and SPDEF binding sites (A549_SPDEF_IP) along with Inputs (A549_NKX2-1_Input and A549_SPDEF_Input) at the locus of *VTCN1* (also known as *B7-H4*). The data of two biological replicates are shown for each ChIP-seq. ChIP-seq was performed using NKX2-1 or SPDEF antibody and chromatin from A549 cells infected with *Nkx2-1-* and/or *SPDEF*-expressing lentivirus as previously reported (Maeda et al, 2012). Chr, chromosome. NKX2-1 bound to the 3' locus of *VTCN1*; however, NKX2-1 did not induce *VTCN1* in A549 cells (Maeda et al, 2012). It is unknown whether NKX2-1 inhibits *VTCN1* due to the absence of *VTCN1* in A549 cells (Klijn et al, 2015). SPDEF did not bind to the locus of *VTCN1* in A549 lung carcinoma cells; however SPDEF bound to the locus of *VTCN1* in MCF7 breast adenocarcinoma cells, suggesting a context-dependent association of SPDEF with the *VTCN1* locus.

B Immunohistochemical analysis indicates that 83% of FOXA3-positive IMA (10 cases) expressed VTCN1 while 17% of FOXA3-positive IMA (2 cases) did not express VTCN1. All of the FOXA3-negative IMA (2 cases) expressed VTCN1. These results suggest that VTCN1 is expressed in human IMA regardless of FOXA3.

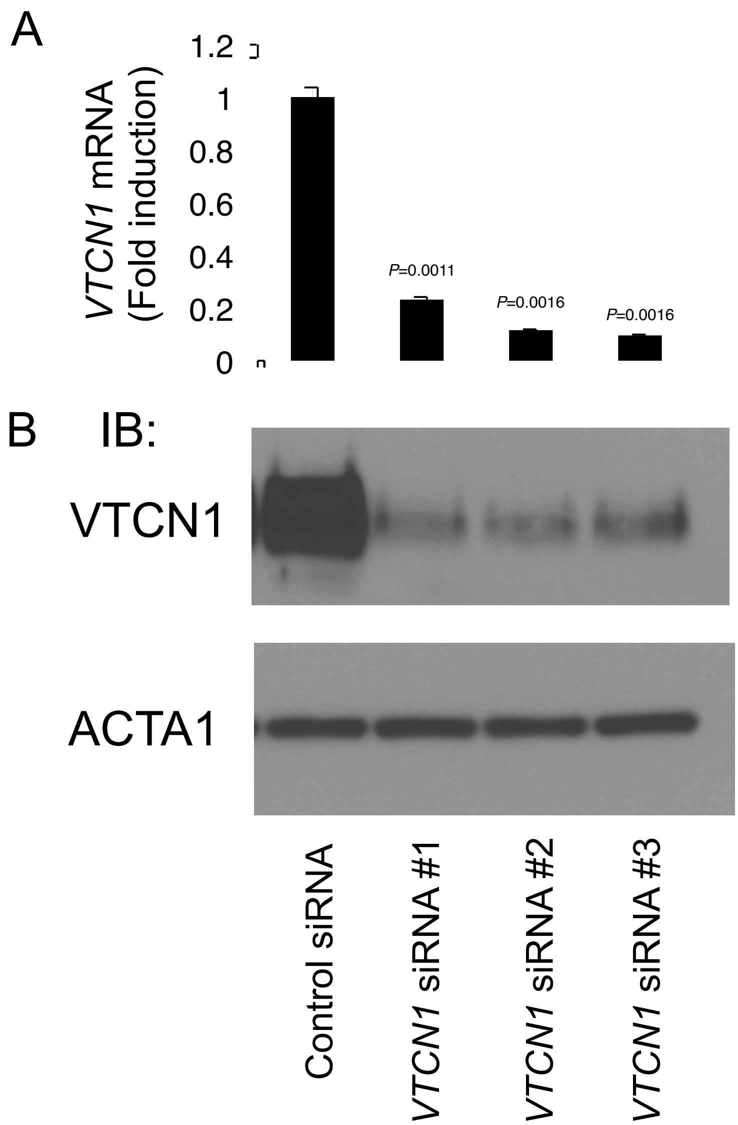

Appendix Figure S3. Endogenous VTCN1 protein detected by immunoblotting using anti-VTCN1 antibody.

A Endogenous *VTCN1* mRNA was reduced by three independent siRNAs targeting *VTCN1*. Three days after transfecting H1437 lung adenocarcinoma cells (expressing endogenous *VTCN1* mRNA according to Klijn et al, 2015) with the siRNAs, RNA was extracted from the cells. The RNA was used for Taqman gene expression qPCR analysis as described in Materials and Methods. Gene expression was normalized by comparison to the constitutive expression of *GAPDH*. Results are expressed as mean ± SEM of experimental triplicates for each group. *P*-value < 0.05 versus control siRNA was considered significant (Student's *t*-test). Two independent experiments were performed.

B Endogenous VTCN1 was reduced by three independent siRNAs targeting *VTCN1*. Three days after the transfection as described above, protein was extracted from the cells. The protein was used for immunoblotting as described in Materials and Methods. Anti-VTCN1 antibody (clone #: D1M8I from Cell Signaling) detects endogenous VTCN1. Constitutively expressed ACTA1 protein was used as a loading control. Shown is a representative image from two independent experiments.
