# Supplementary material for: Gene signature driving invasive mucinous adenocarcinoma of the lung
Source: EMBO Mol Med. 2017 Mar 2;9(4):462–81. doi: 10.15252/emmm.201606711 (PMC5376761; doi:10.15252/emmm.201606711)
Supplement: Supplementary file 2 — Expanded View Figures PDF [file EMMM-9-462-s002.pdf]

Expanded View Figures

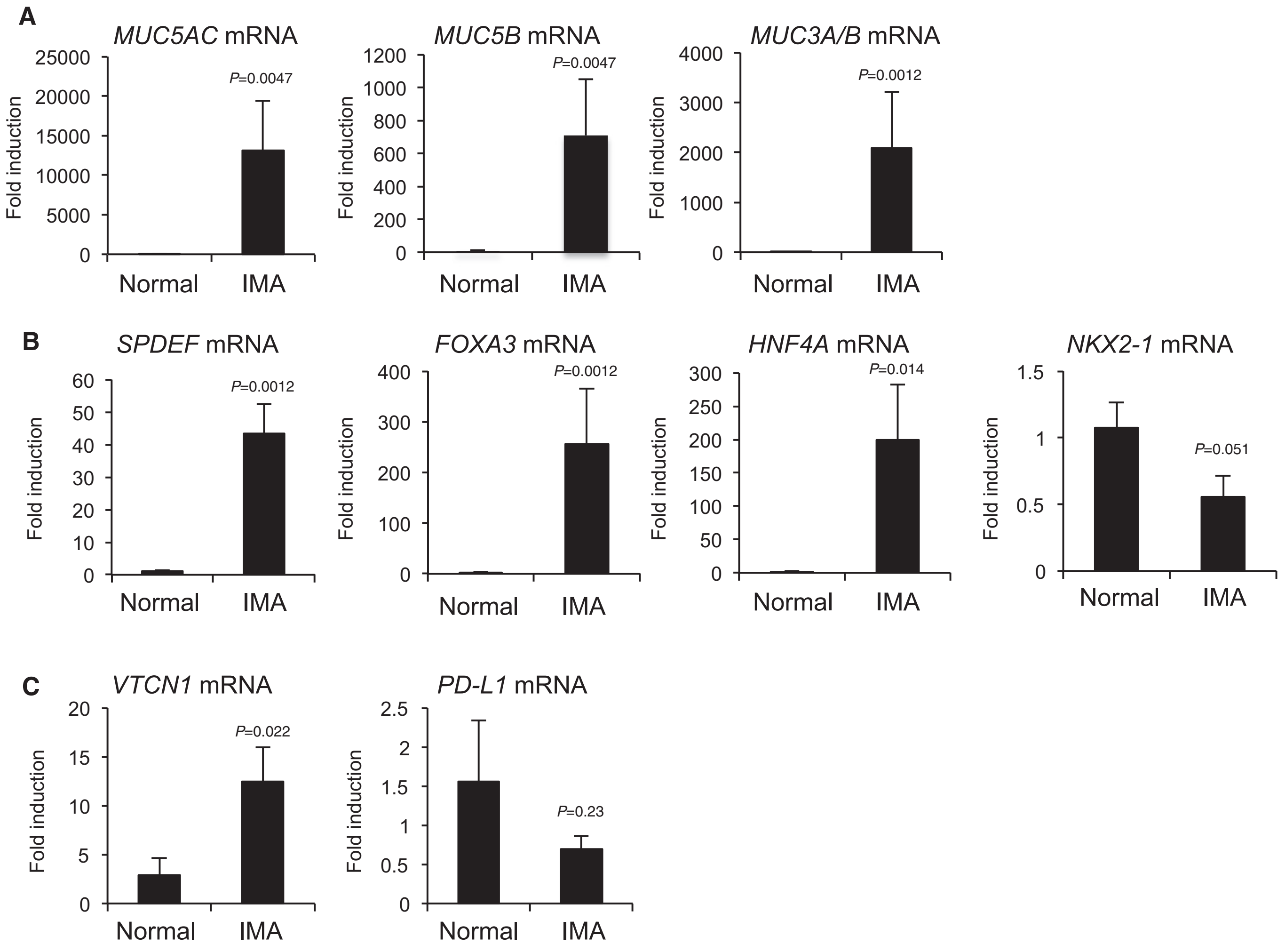

Figure EV1. Taqman qPCR validation of differentially regulated genes in human IMA compared to normal lung tissues.

A Mucin genes (*MUC5AC*, *MUC5B*, and *MUC3A/B*) were significantly induced in human IMA (n = 7) compared to normal lung tissues (n = 6).
B Transcription factors *SPDEF*, *FOXA3*, and *HNF4A* but not *NKX2-1* were significantly induced in human IMA (n = 7) compared to normal lung tissues (n = 6).
C Immune checkpoint gene *VTCN1* but not *PD-L1* was significantly induced in human IMA (n = 7) compared to normal lung tissues (n = 6).

Data information: See patient information in Dataset EV1. Taqman gene expression qPCR analysis was performed as described in Materials and Methods. Each gene expression was normalized by comparison with the constitutive expression of *ACTB* (*GAPDH* was not used for normalization since *GAPDH* was induced in human IMA compared to normal lung tissue; see Dataset EV2). Results are expressed as mean ± SEM of biological replicates for each group. $P < 0.05$ versus normal was considered significant (Mann–Whitney test).

© 2017 The Authors

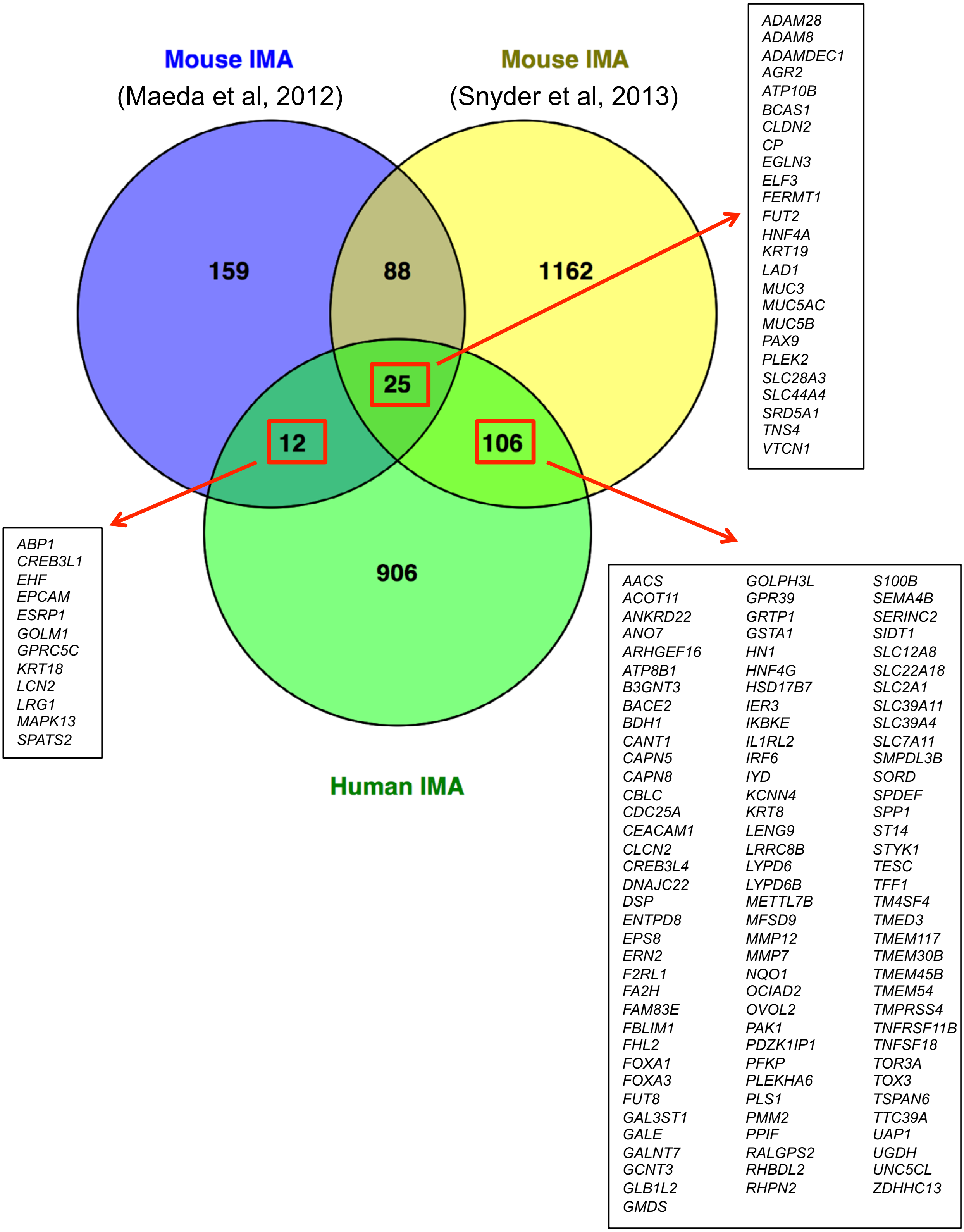

Figure EV2. 143 genes that were commonly expressed in both mouse and human IMA constitute the Mucinous Lung Tumor Signature.
Shown are the 143 genes induced in the two IMA mouse models (Maeda *et al*, 2012; Snyder *et al*, 2013) and human IMA.

© 2017 The Authors

Figure EV3. FOXA3 or SPDEF drives mucinous lung tumors in KRAS^{G12D} lung tumor mouse model.

A Shown are schemes for the conditional lung cancer mouse model (upper panel) and the timing of doxycycline administration (lower panel). Rat *Scgb1a1* (also known as CCSP) promoter (line 2) is active in mouse Club (also known as Clara) and alveolar type II cells (Perl *et al*, 2009). Doxycycline induces FOXA3 or SPDEF along with KRAS^{G12D} only in lung epithelial Club and alveolar type II cells.

B FOXA3 (left panel) or SPDEF (right panel) along with KRAS^{G12D} induced mucinous lung tumors *in vivo*. SPDEF along with KRAS^{G12D} induced malignant lung tumors (tubulopapillary-like carcinoma). AAH: atypical adenomatous hyperplasia (a putative precursor lesion of adenocarcinoma of the lung). The tumor criteria are modified from Sutherland *et al* (2014). Percentage of tumor types was obtained by analyzing number and types of lung tumors on each lung section from five different mice in each group. $P < 0.05$ versus *Scgb1a1-rtTA;[tetO]-Kras*^{G12D} was considered significant (Student's *t*-test).

C Transgenic mice expressing KRAS^{G12D} and FOXA3 (*Scgb1a1-rtTA;[tetO]-Kras*^{G12D}*;[tetO]-Foxa3*) in lung epithelium survived significantly longer than mice expressing only KRAS^{G12D} (*Scgb1a1-rtTA;[tetO]-Kras*^{G12D}; left panel), while mice expressing KRAS^{G12D} and SPDEF (*Scgb1a1-rtTA;[tetO]-Kras*^{G12D}*;[tetO]-Spdef*) in lung epithelium survived significantly shorter than mice expressing only KRAS^{G12D} (*Scgb1a1-rtTA;[tetO]-Kras*^{G12D}; right panel). Kaplan–Meier survival analysis was performed using Prism 6. Statistical significance was obtained by log-rank (Mantel–Cox) test. $P < 0.05$ versus *Scgb1a1-rtTA;[tetO]-Kras*^{G12D} was considered significant. See Dataset EV6 for further details.

© 2017 The Authors

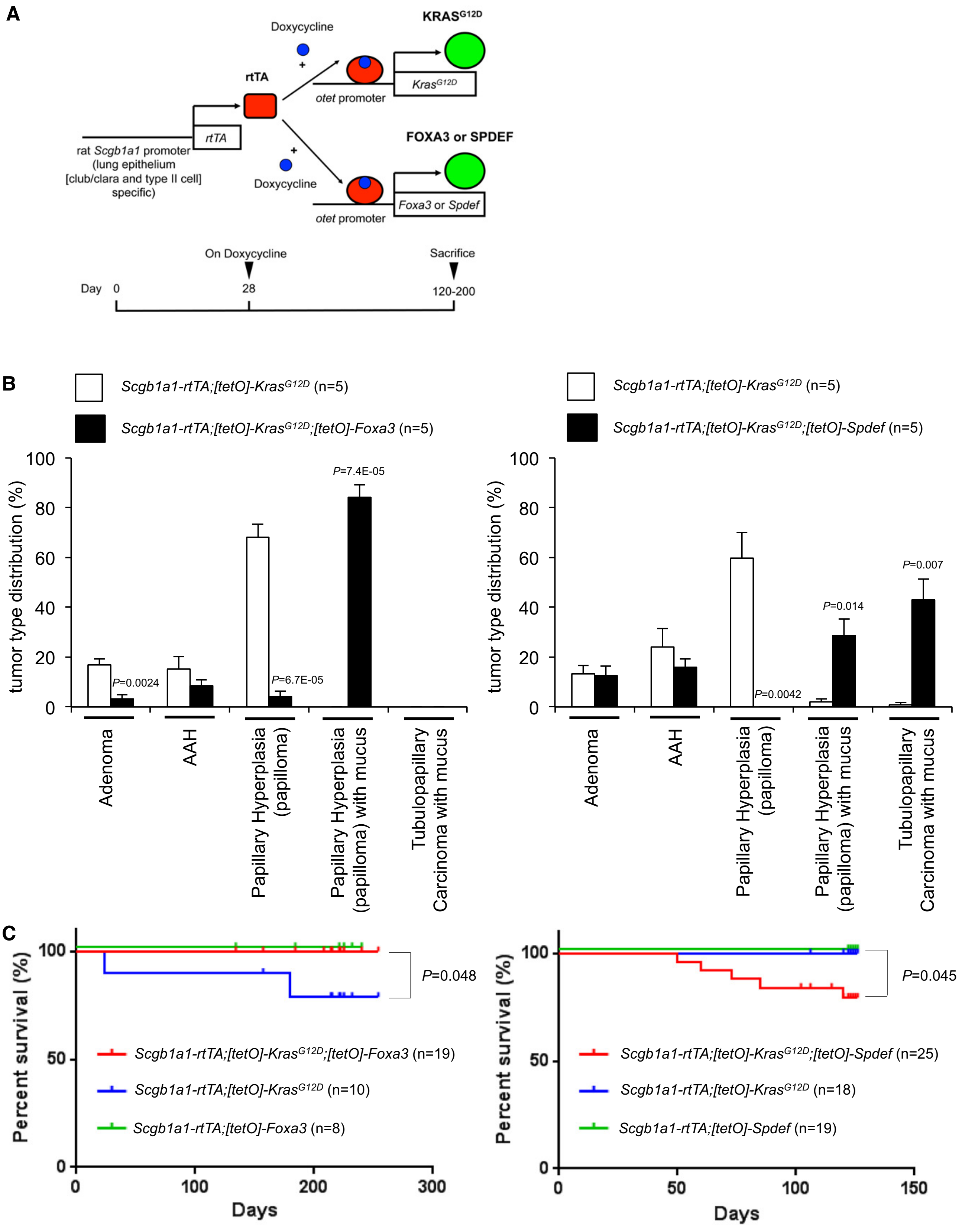

Figure EV3.

 © 2017 The Authors

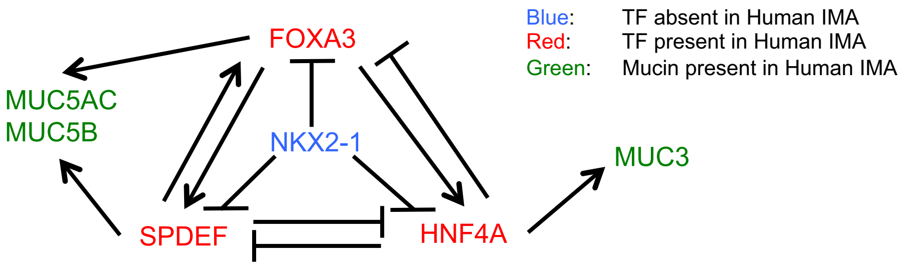

Figure EV4. Regulation of transcription factors (TF) involved in mucin gene expression in human IMA.

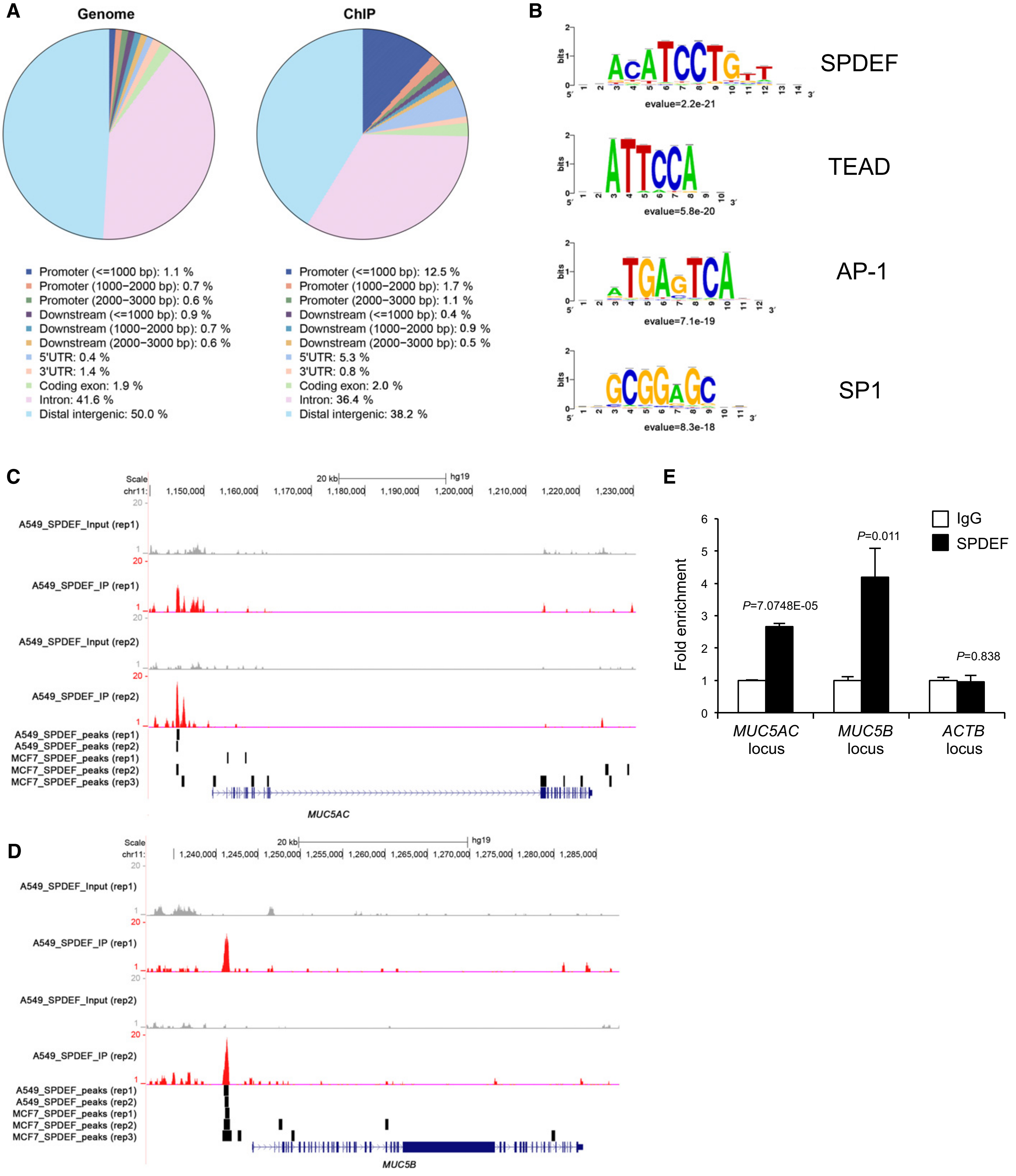

Figure EV5.

 © 2017 The Authors

Figure EV5. ChIP-seq analysis identifying SPDEF binding sites in A549 human lung carcinoma cells.

A Distribution of SPDEF binding sites in genome (genome in general vs. SPDEF ChIP). SPDEF binding sites are located in promoter and 5′-UTR.

B Most significantly present transcription factor-binding DNA motifs in SPDEF ChIP-seq sites. SPDEF binding DNA motif was identified. Significance was measured by "evalue", adjusted *P*-value generated by RSAT peak-motifs (Thomas-Chollier *et al*, 2012a,b).

C Shown is a UCSC genome browser view of ChIP-seq indicating SPDEF binding sites (A549-SPDEF_IP) along with Input (A549_SPDEF_Input) at the locus of *MUC5AC*. The data of two biological replicates are shown. ChIP-seq was performed using SPDEF antibody and chromatin from A549 cells infected with *SPDEF*-expressing lentivirus as described in Materials and Methods. Chr, chromosome.

D UCSC genome browser view is shown as described in (C) except ChIP-seq indicating SPDEF binding sites (A549_SPDEF_IP) along with Input (A549_SPDEF_Input) at the locus of *MUC5B*. ChIP-seq was performed as described above. Chr, chromosome.

E ChIP-qPCR showing SPDEF or IgG fold enrichment in A549 cells infected with *SPDEF*-expressing lentivirus as described in Materials and Methods. Results are expressed as mean ± SEM of experimental triplicates for each group. $P < 0.05$ versus IgG control was considered significant (Student's *t*-test). Two independent experiments were performed. The SPDEF binding to the loci of *MUC5AC* and *MUC5B* but not that of *ACTB* was confirmed.

© 2017 The Authors
