# Supplementary figures and images for: Gene signature driving invasive mucinous adenocarcinoma of the lung
Source: EMBO Mol Med. 2017 Mar 2;9(4):462–81. doi: 10.15252/emmm.201606711 (PMC5376761; doi:10.15252/emmm.201606711)

Gel images for Fig 4C

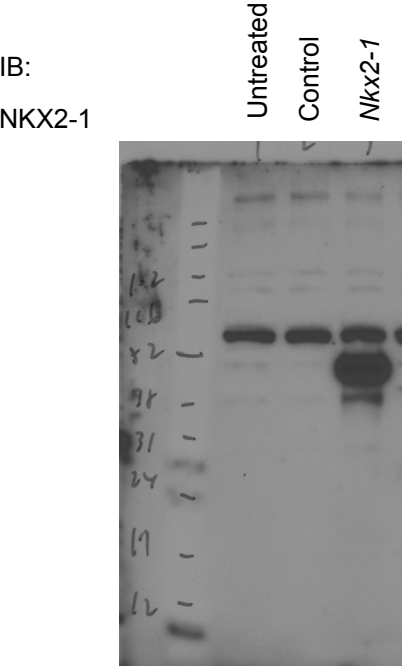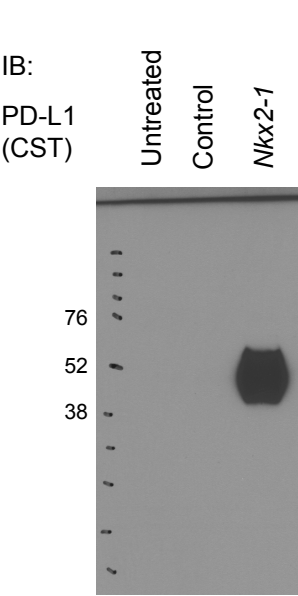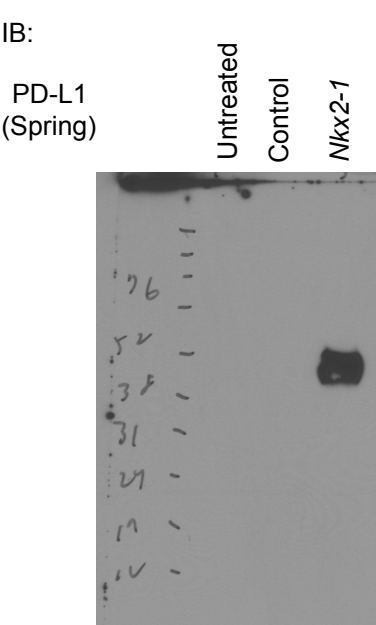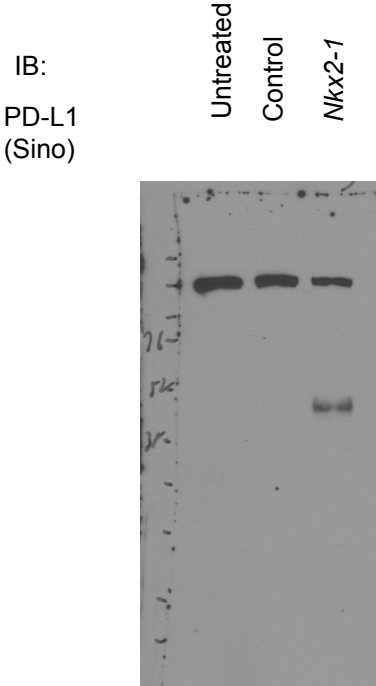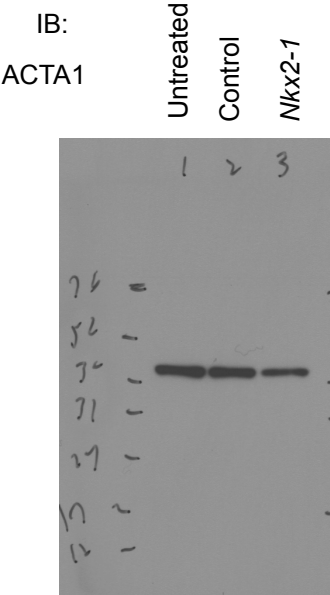

Gel images for Fig 4D

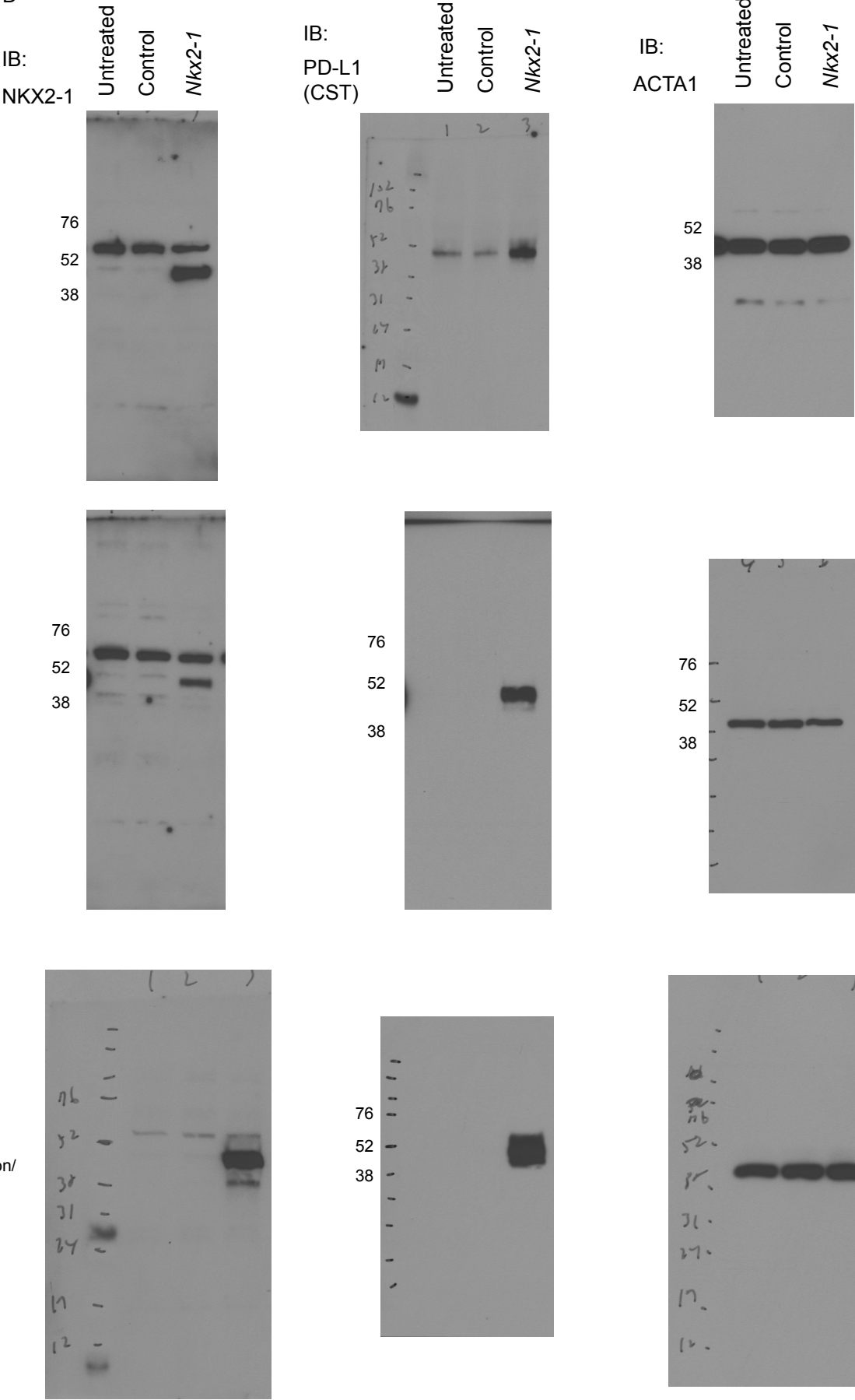

Supplement: Supplementary file 12 — Source Data for Figure 4 [file EMMM-9-462-s011.pdf]

Gel images for Fig 6A

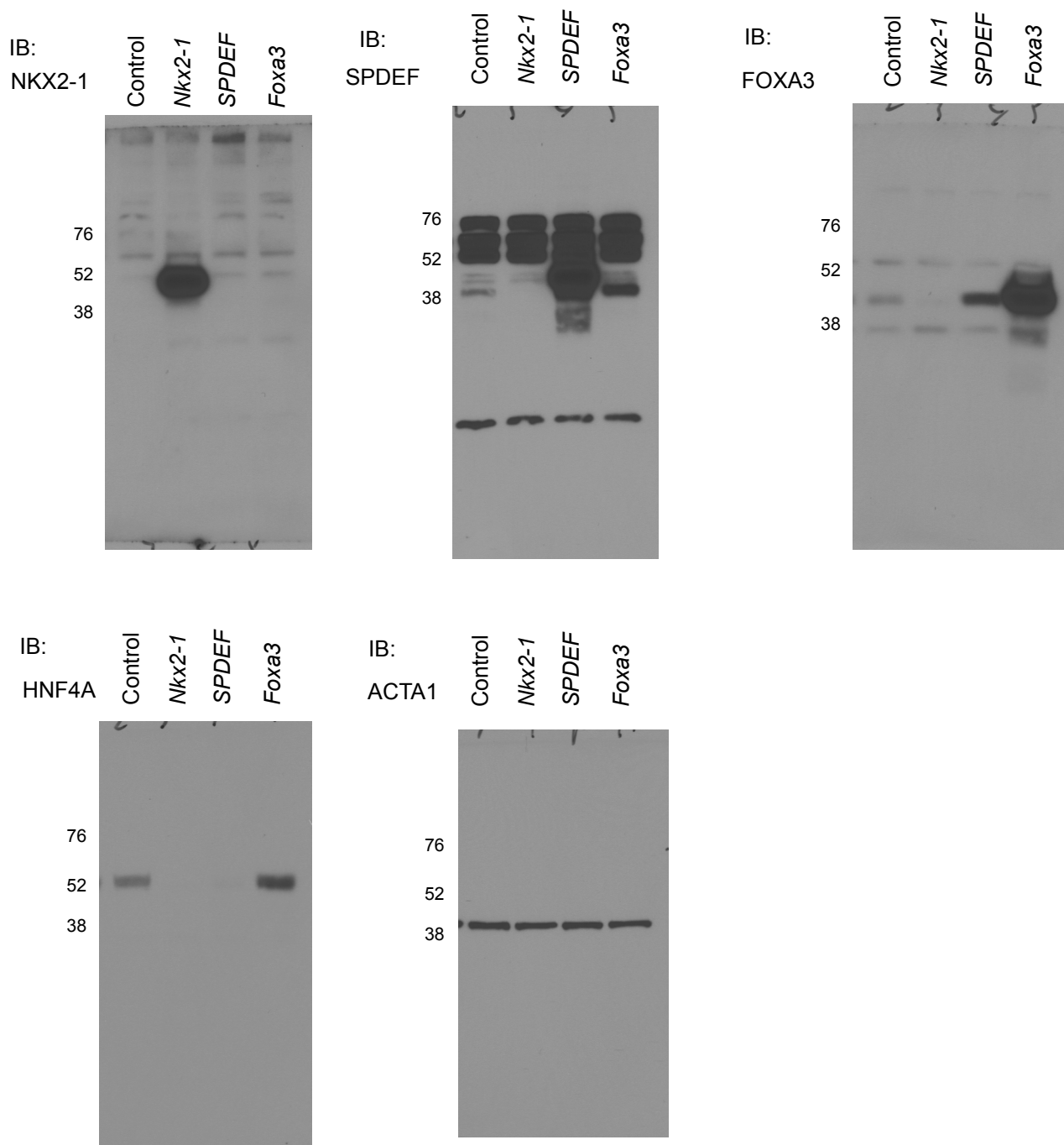

Fig 6

Gel images for Fig 6E

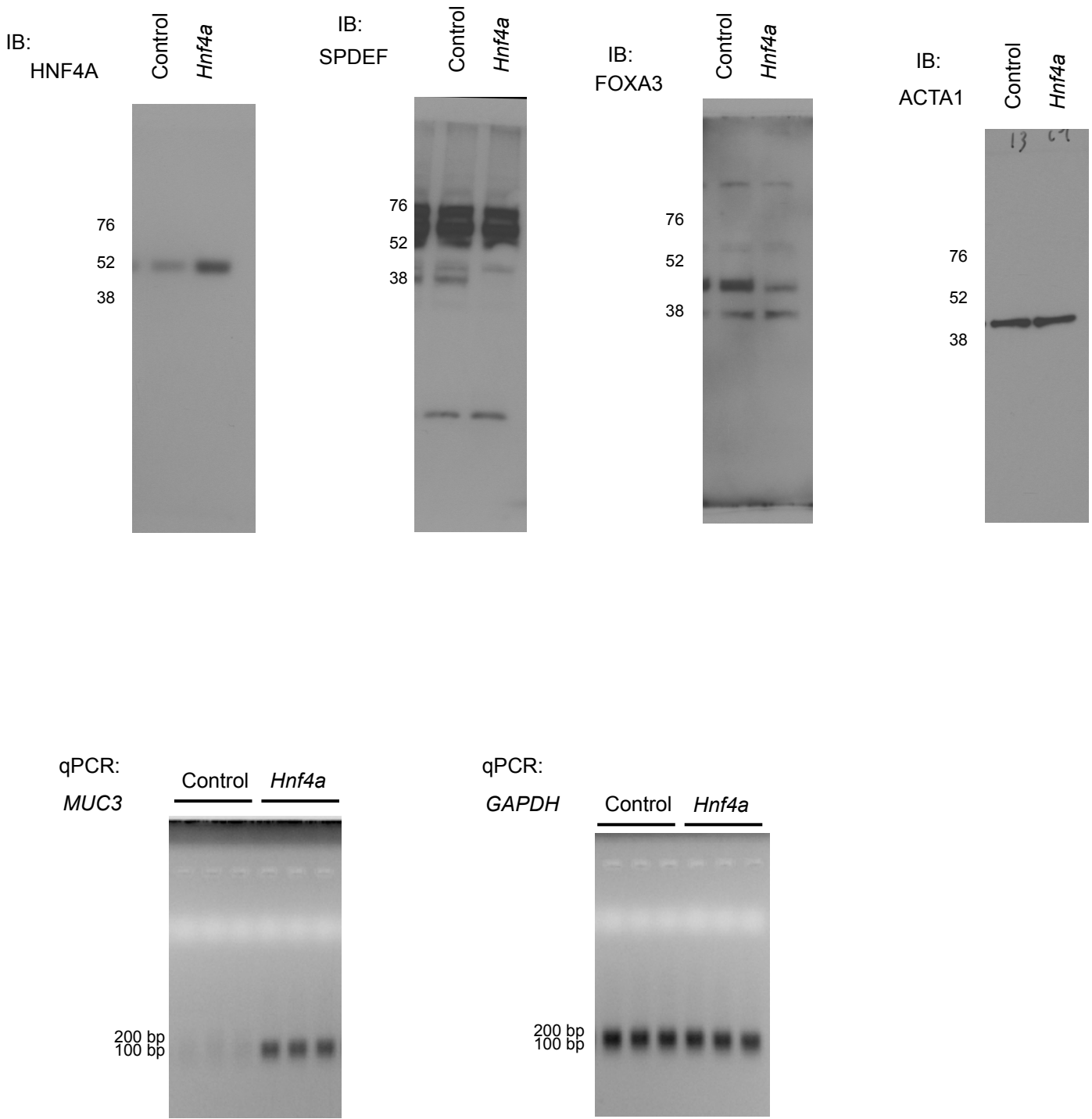

Supplement: Supplementary file 13 — Source Data for Figure 6 [file EMMM-9-462-s012.pdf]
